# Transcriptome-wide association study identified candidate genes associated with gut microbiota

**DOI:** 10.1186/s13099-021-00474-w

**Published:** 2021-12-18

**Authors:** Chuyu Pan, Yujie Ning, Yumeng Jia, Shiqiang Cheng, Yan Wen, Xuena Yang, Peilin Meng, Chun’e Li, Huijie Zhang, Yujing Chen, Jingxi Zhang, Zhen Zhang, Feng Zhang

**Affiliations:** Key Laboratory of Trace Elements and Endemic Diseases of National Health and Family Planning Commission, National Health Commission of the People’s Republic of China, School of Public Health, Health Science Center, Xi’an Jiaotong University, Xi’an, 71006 China

**Keywords:** Gut microbiota, Transcriptome-wide association study (TWAS), Genome-wide association study (GWAS), Pathway

## Abstract

**Background:**

Gut microbiota is closely associated with host health and disease occurrence. Host genetic factor plays an important role in shaping gut microbial communities. The specific mechanism of host-regulated gene expression affecting gut microbiota has not been elucidated yet. Here we conducted a transcriptome-wide association study (TWAS) for gut microbiota by leveraging expression imputation from large-scale GWAS data sets.

**Results:**

TWAS detected multiple tissue-specific candidate genes for gut microbiota, such as FUT2 for genus *Bifidobacterium* in transverse colon (*P*_PERM.ANL_ = 1.68 × 10^–3^) and SFTPD for an unclassified genus of *Proteobacteria* in transverse colon (*P*_PERM.ANL_ = 5.69 × 10^–3^). Fine mapping replicated 3 candidate genes in TWAS, such as HELLS for *Streptococcus* (PIP = 0.685) in sigmoid colon, ANO7 for *Erysipelotrichaceae* (PIP = 0.449) in sigmoid colon. Functional analyses detected 94 significant GO terms and 11 pathways for various taxa in total, such as GO_NUCLEOSIDE_DIPHOSPHATASE_ACTIVITY for *Butyrivibrio* (FDR *P* = 1.30 × 10^–4^), KEGG_RENIN_ANGIOTENSIN_SYSTEM for *Anaerostipes* (FDR *P* = 3.16 × 10^–2^). Literature search results showed 12 genes prioritized by TWAS were associated with 12 diseases. For instance, SFTPD for an unclassified genus of *Proteobacteria* was related to atherosclerosis, and FUT2 for *Bifidobacterium* was associated with Crohn’s disease.

**Conclusions:**

Our study results provided novel insights for understanding the genetic mechanism of gut microbiota, and attempted to provide clues for revealing the influence of genetic factors on gut microbiota for the occurrence and development of diseases.

**Supplementary Information:**

The online version contains supplementary material available at 10.1186/s13099-021-00474-w.

## Background

Gut microbiota is an enormous and complex ecosystem, which is closely associated with the host by affecting metabolism, immunity and other physiological functions [1, 2]. Numerous studies have suggested that the correlation of gut microbiota with the incidence of complex diseases. A case–control study showed the microbial pattern of women with breast cancer is different from healthy women in terms of bacterial type, relative abundance and function [3]. A cohort study of Indian Children found that the proportion of *Firmicutes* in Autistic Spectrum Disorder (ASD) children was higher than healthy children [4]. In addition, the gut microbiota might involve in modulation of body mass index and blood lipid level according to the LifeLines-DEEP population cohort study which consists of 893 subjects [5]. However, the mechanism of a large part of diseases induced by gut microbiota is still unclear, needing further research to elucidate.

The composition of the gut microbiota is shaped by multiple factors including environment, diet, medication as well as internal parameters [6]. In recent decades, great deal of evidence has indicated that host genetic factor plays indispensable role in shaping the gut microbial communities. Lim et al*.* found monozygotic twin pairs had more similar gut microbial communities compared with other family members, and 50 gut microbial taxa (58.8%) showed significant heritability among the 85 taxa identified with heritability estimates valued ranging between 13.1% and 45.7% [7]. Additionally, based on a large (n = 645) mouse advanced intercross line, microbial quantitative trait loci (mbQTLs) could significantly affect gut microbial taxa [8]. Moreover, microbial genome-wide association analysis (mGWAS) has been conducted in recent years to reveal loci related to the gut microbiota. According to a previous study, *Lactococcus* bacteria could be affected by single nucleotide polymorphism (SNP) rs2294239 in ZNRF3 gene, which is associated with body fat distribution [9].

The gut microbiota can be regarded as a trait affected by genetic factors [8]. Although GWAS has contributed to a great number of genetic clues related to complex diseases and traits, it has limitation in explaining how the genetic variations regulate gene expression alone because the SNPs identified mainly located in non-coding regions [10]. In recent years, expression quantitative trait loci (eQTLs) have been widely used to elucidate the influence of genetic variants at gene expression level [11]. Subsequently, integrated analysis of GWAS and eQTLs became practical in exploring the effect of gene expression on complex traits [12]. One such family of methods is transcriptome-wide association study (TWAS), which was conducted to impute expression from genetic data, showing great power to prioritize candidate genes of complex traits interested, and has been used to identify the associations between many diseases and genes [13]. For example, Liao et al. identified KAT2B and TMEM161B as causal genes for attention deficit hyperactivity disorder by TWAS [14]. Another TWAS detected 25 genes, including CELA3B, whose predictive expression was statistically significantly associated with pancreatic cancer risk [15]. To the best of our knowledge, no TWAS was applied in gut microbiota study until now.

In this study, we performed TWAS analysis and fine mapping of gut microbiota for multiple tissues by leveraging expression imputation from large-scale GWAS data sets. Subsequently, functional analysis was conducted for exploration of the biological functions and pathways of significant gene sets. Furthermore, we sorted out diseases associated with gut microbiota candidate genes by manually reviewing the literature.

## Methods

### mGWAS of gut microbiota

The human microbiota GWAS summary data were obtained from a study published by Hughes et al. [16]. The study projects consisted of 2223 individuals from the Flemish Gut Flora Project (FGFP) cohort. DNA was extracted from frozen fecal samples and used for 16S ribosomal RNA gene sequencing subsequently. Among 499 taxon-derived abundances in FGFP, 92 taxa met the analysis criteria, which were identified independent phenotypes. The presence/absence (P/A) phenotype (binary) and the zero-truncated (all zero values set as missing) abundance (AB) phenotype (continuous) were generated for taxa where > 5% of individuals in FGFP had an abundance measurement of zero. The genome-wide genotyping of FGFP was conducted using either the Human Core Exome v.1.0 array or the Human Core Exome v.1.1 array. Snptest.2.5.0 was used for association analysis. In brief, 157 microbial traits, including 62 presence/absence (P/A-HB) and 95 in abundance (AB-RNT) microbial phenotypes were included in the subsequent analysis. Detailed information on subjects, study design, statistical analysis and quality control can be found in the publication [16].

### TWAS of gut microbiota

TWAS of gut microbiota was performed by FUSION software, which precomputed the gene expression weights of various tissues using a small set of individuals with both gene expression and genotype data. The *cis-*genetic component of expression was then imputed into much larger sets of phenotyped individuals according to SNP genotype data. In this study, we used Bayesian Sparse Linear Mixed Model (BSLMM) to calculate the SNP expression weight of a gene's 1-Mb *cis* loci [17]. Let w denotes the weights. Z denotes the scores of gut microbiota. L denotes the SNP-correlation matrix. The association testing statistics between predicted gene expression and each taxon was calculated as ${Z}_{TWAS}=w{{\prime}}Z/\left({w}^{{\prime}}Lw\right)1/2$. The imputed expression data can be regarded as a linear model of genotypes with weights based on the correlation between gene expression and SNPs in the training data, linkage disequilibrium (LD) among SNPs was considered [13]. Finally, the association between target traits and the expression level of genes was estimated by integrating analysis of mGWAS summary data with gene expression weights. The precomputed expression weights of tissues derived from the genotype-Tissue expression (GTEx) project were downloaded from FUSION websites (http://gusevlab.org/projects/fusion/). Specific in this study, we used the sigmoid colon and transverse colon as reference panels. Following the recommendation in FUSION software [13], we generated the cleaned mGWAS summary statistics data by leverage LD reference panel for further analyses, and the mGWAS summary statistics have not been trimmed or thresholded before. The percentage of SNPs in the LD reference available in the FGFP mGWAS data was approximately 13.8% for each microbial trait. We implemented 2000 permutation tests for each FUSION analysis to reduce the inflation of by-chance QTL co-localization. In this study, the analytical permutation *P* value (*P*_PERM.ANL_) < 0.05 were considered to be significant.

### TWAS fine mapping

Fine-mapping of causal gene sets (FOCUS) approach was performed for prioritizing genes with strong evidence for causality in TWAS analyses [18]. FOCUS integrates GWAS summary data and expression prediction weights estimated from the eQTL reference panel, considering the LD of all SNPs in the risk region, and finally estimates the probability (probability estimates of causality, PIP) of any given gene set to explain the TWAS signal [18] for each gene. The gene included in 90%-credible set is more likely to be causal than any other gene in the region. Consistently with TWAS analyses, the transverse colon and sigmoid colon were used as the reference panels in FOCUS analysis. The threshold for screening of mGWAS summary data was 1 × 10^–5^ [16].

### Functional analyses

The gut microbiota related genes identified by TWAS (*P*_PERM.ANL_ < 0.05) were used for functional analyses by Functional Mapping and Annotation (FUMA) online platform [19]. *P* values were calculated by FUMA for each Gene Ontology (GO) term and pathway. The FDR *P* value < 0.05 was considered as significant.

### Verification of gene and disease association

The literature mining was performed to show the lists of diseases related to the genes. The PubMed (https://pubmed.ncbi.nlm.nih.gov/) was searched to identify whether the significant genes of each taxon identified by TWAS were the causal gene of the target diseases.

## Results

### TWAS results

In total, the TWAS of 157 microbial traits were performed by FUSION. In presence/absence (P/A-HB) phenotype, 1693 genes were identified by TWAS for overall 62 microbial traits (Additional file 1: Table S1, Additional file 2: Table S2, Additional file 3: Table S3), such as TOB2P1 for *Enterococcaceae* in sigmoid colon (*P*_PERM.ANL_ = 1.94 × 10^–50^), KCNIP3 for *Veillonellaceae* in transverse colon (*P*_PERM.ANL_ = 8.35 × 10^–33^), WDR6 for *Coprococcus* in sigmoid colon (*P*_PERM.ANL_ = 1.1 × 10^–16^). Accordingly, 2247 genes were detected for 95 microbial traits in abundance (AB-RNT) phenotype, such as WDR6 for *Butyrivibrio* in sigmoid colon (*P*_PERM.ANL_ = 1.24 × 10^–64^), FBXO41 for *Clostridium* XlVa in transverse colon (*P*_PERM.ANL_ = 1.47 × 10^–21^), CENPE for *Veillonellaceae* in sigmoid colon (*P*_PERM.ANL_ = 2.30 × 10^–17^). Table 1 summarizes the top 20 significant genes associated with microbiota in two phenotypes, respectively.

**Table 1:** Top 20 candidate genes detected by TWAS in P/A and AB models

Gene	Tissue	Microbiota trait	Z	*P*
P/A-HB phenotype				
DNAJC9-AS1	Transverse colon	G_Ruminococcus	− 2.28	4.66 × 10^–154^
CCDC36	Sigmoid colon	F_Enterococcaceae	− 2.22	6.46 × 10^–130^
RP11-804H8.6	Sigmoid colon	F_Enterobacteriaceae	2.02	1.19 × 10^–55^
TOB2P1	Sigmoid colon	F_Enterococcaceae	2.02	1.94 × 10^–50^
ARHGAP1	Transverse colon	C_Gammaproteobacteria	1.96	4.85 × 10^–44^
RP11-697N18.3	Transverse colon	F_Peptostreptococcaceae	2.54	3.91 × 10^–33^
KCNIP3	Transverse colon	F_Veillonellaceae	1.97	8.35 × 10^–33^
C3orf18	Sigmoid colon	C_Deltaproteobacteria	− 2.34	3.61 × 10^–27^
C3orf18	Sigmoid colon	F_Enterobacteriaceae	− 2.07	5.68 × 10^–22^
AC011330.5	Sigmoid colon	G_Clostridium_sensu_stricto	− 2.19	3.47 × 10^–21^
RNF138P1	Sigmoid colon	G_Gemmiger	2.029	1.56 × 10^–19^
ARIH2	Transverse colon	G_Coprococcus	2.57	3.39 × 10^–18^
ELMO3	Transverse colon	G_Collinsella	− 2.33	1.02 × 10^–16^
RP11-344N10.5	Sigmoid colon	G_Bifidobacterium	− 2.04	1.09 × 10^–16^
WDR6	Sigmoid colon	G_Coprococcus	− 2.07	1.10 × 10^–16^
CYP1A1	Transverse colon	G_Paraprevotella	2.30	2.60 × 10^–15^
PROM2	Sigmoid colon	G_F_Coriobacteriaceae	2.65	1.74 × 10^–13^
CYP1A1	Transverse colon	G_F_Porphyromonadaceae	2.28	9.01 × 10^–13^
FBXO41	Transverse colon	G_Lactococcus	− 2.44	4.30 × 10^–12^
PROM2	Sigmoid colon	G_F_Rhodospirillaceae	2.07	3.46 × 10^–11^
AB-RNT phenotype				
RP3-462E2.5	Transverse colon	G_Sporobacter	2.243	3.53 × 10^–98^
WDR6	Sigmoid colon	G_Butyrivibrio	2.50	1.24 × 10^–64^
ARIH2	Transverse colon	G_P_Proteobacteria	− 3.33	2.18 × 10^–63^
TOB2P1	Sigmoid colon	G_Ruminococcus2	2.01	2.72 × 10^–29^
IGKV6-21	Transverse colon	G_Collinsella	2.16	3.56 × 10^–26^
C3orf18	Transverse colon	F_Enterobacteriaceae	− 2.77	3.09 × 10^–24^
C3orf18	Sigmoid colon	C_Gammaproteobacteria	− 2.00	4.03 × 10^–22^
FBXO41	Transverse colon	G_Clostridium_XlVa	− 2.60	1.47 × 10^–21^
IGKV6-21	Transverse colon	G_P_Bacteroidetes	− 2.16	3.58 × 10^–21^
RP11-10C24.3	Sigmoid colon	G_Sutterella	2.97	1.07 × 10^–19^
C3orf18	Transverse colon	C_Gammaproteobacteria	− 2.14	7.08 × 10^–19^
ZNF33A	Transverse colon	G_P_Bacteroidetes	2.50	8.09 × 10^–19^
SLC33A1	Sigmoid colon	G_Sutterella	− 2.28	1.10 × 10^–17^
DNAJB12	Transverse colon	P_Proteobacteria	2.59	1.19 × 10^–17^
CENPE	Sigmoid colon	F_Veillonellaceae	2.20	2.30 × 10^–17^
MUTYH	Transverse colon	G_Prevotella	− 2.39	2.74 × 10^–17^
RP11-365H22.2	Transverse colon	O_Burkholderiales	2.08	3.75 × 10^–16^
SIL1	Transverse colon	G_Streptococcus	2.31	1.10 × 10^–15^
GINM1	Sigmoid colon	G_F_Lachnospiraceae	2.50	2.80 × 10^–15^
RP11-365H22.2	Transverse colon	G_P_Firmicutes	1.97	2.26 × 10^–14^

**Fig. 1:**
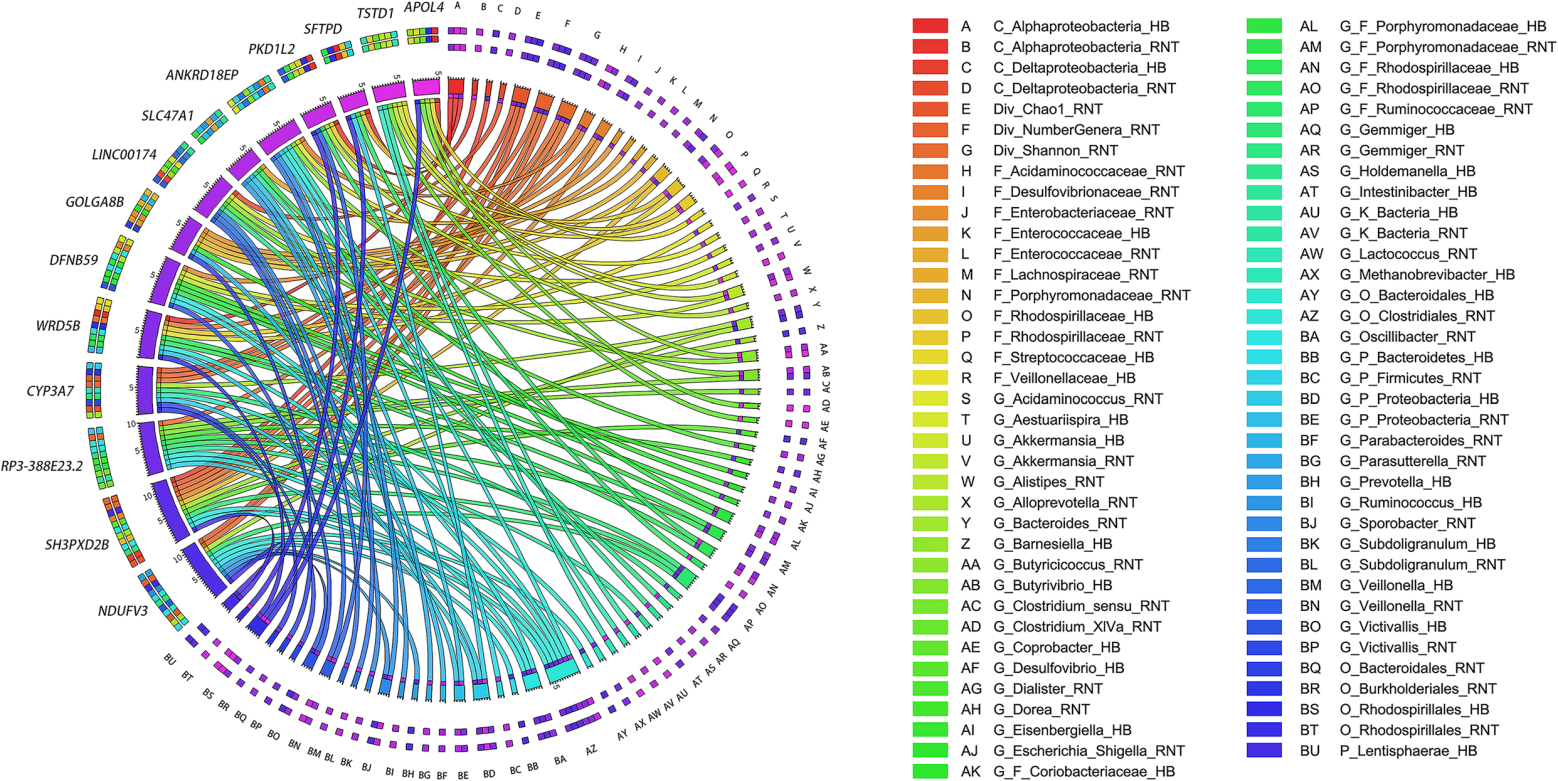
Top 14 overlapped candidate genes with the most repetitions in all microbial traits. Circos shows the top 14 candidate genes with the most repeats of all gut microbiota in transverse colon and sigmoid colon. The associations for each OTU with multiple genes are also exhibited. The labels on the left of the figure represent gene names, and the labels on the right are sorted alphabetically, representing different OTUs

We summarized overlapped candidate genes for different microbial traits (Fig. 1, Additional file 4: Table S4), such as NDUFV3 for *Lentisphaerae* (HB), *Bacteroidales* (HB), *Prevotella* (HB), an unclassified genus of order *Clostridiales* (RNT), an unclassified genus of family *Ruminococcacea* (RNT), *Victivallis* (HB), *Bacteroides* (RNT), *Sporobacter* (RNT), an unclassified genus of phylum *Bacteroidetes* (HB), Chao diversity (RNT) and the number of genera observed (RNT); and SFTPD gene for *Rhodospirillaceae* (HB), *Alphaproteobacteria* (HB), an unclassified genus of phylum *Proteobacteria* (HB), *Rhodospirillales* (HB) and an unclassified genus of family *Rhodospirillaceae* (HB). Table 2 shows top 6 genes with the most repeats for microbial traits.

**Table 2:** Top 6 overlapped candidate genes for different microbial traits

Gene	Tissue	Microbial trait	Z	*P*
NDUFV3	Sigmoid colon	P_Lentisphaerae_HB	3.61	1.35 × 10^–3^
	Sigmoid colon	Div_NumberGenera_RNT	3.52	1.58 × 10^–3^
	Sigmoid colon	G_O_Bacteroidales_HB	3.45	1.96 × 10^–3^
	Sigmoid colon	G_Prevotella_HB	3.25	1.99 × 10^–3^
	Sigmoid colon	G_O_Clostridiales_RNT	3.33	2.01 × 10^–3^
	Sigmoid colon	Div_Chao1_RNT	3.18	2.48 × 10^–3^
	Transverse colon	P_Lentisphaerae_HB	3.41	2.51 × 10^–3^
	Transverse colon	G_O_Clostridiales_RNT	3.20	3.19 × 10^–3^
	Sigmoid colon	G_F_Ruminococcaceae_RNT	3.35	3.65 × 10^–3^
	Sigmoid colon	G_Victivallis_HB	3.08	4.37 × 10^–3^
	Transverse colon	G_O_Bacteroidales_HB	2.98	4.64 × 10^–3^
	Sigmoid colon	G_Bacteroides_RNT	− 3.22	5.11 × 10^–3^
	Transverse colon	G_Bacteroides_RNT	− 3.30	6.08 × 10^–3^
	Sigmoid colon	G_Sporobacter_RNT	3.26	1.01 × 10^–2^
	Sigmoid colon	G_P_Bacteroidetes_HB	2.85	1.14 × 10^–2^
SH3PXD2B	Sigmoid colon	Div_Shannon_RNT	− 4.04	1.26 × 10^–3^
	Sigmoid colon	Div_Chao1_RNT	− 3.54	1.44 × 10^–3^
	Sigmoid colon	G_F_Ruminococcaceae_RNT	− 3.49	1.60 × 10^–3^
	Sigmoid colon	F_Desulfovibrionaceae_RNT	− 3.54	1.87 × 10^–3^
	Sigmoid colon	F_Porphyromonadaceae_RNT	− 4.02	2.04 × 10^–3^
	Sigmoid colon	Div_NumberGenera_RNT	− 3.46	2.58 × 10^–3^
	Sigmoid colon	G_Desulfovibrio_HB	− 3.42	2.64 × 10^–3^
	Sigmoid colon	C_Deltaproteobacteria_HB	− 3.16	3.33 × 10^–3^
	Sigmoid colon	G_O_Clostridiales_RNT	− 3.55	4.15 × 10^–3^
	Sigmoid colon	G_Barnesiella_HB	− 3.50	4.77 × 10^–3^
	Sigmoid colon	P_Lentisphaerae_HB	− 3.47	5.03 × 10^–3^
	Sigmoid colon	G_Oscillibacter_RNT	− 3.72	6.04 × 10^–3^
RP3-388E23.2	Transverse colon	G_Butyrivibrio_HB	3.93	3.46 × 10^–5^
	Transverse colon	Div_Shannon_RNT	3.43	4.13 × 10^–4^
	Transverse colon	G_O_Clostridiales_RNT	2.70	1.33 × 10^–3^
	Transverse colon	G_Clostridium_sensu_RNT	2.71	2.73 × 10^–3^
	Transverse colon	G_F_Ruminococcaceae_RNT	2.72	2.94 × 10^–3^
	Sigmoid colon	G_F_Ruminococcaceae_RNT	2.61	5.84 × 10^–3^
	Sigmoid colon	G_F_Porphyromonadaceae_RNT	2.56	7.77 × 10^–3^
	Sigmoid colon	G_Dorea_RNT	2.06	1.04 × 10^–2^
	Transverse colon	G_P_Proteobacteria_HB	2.43	1.21 × 10^–2^
	Sigmoid colon	G_Intestinibacter_HB	2.08	1.98 × 10^–2^
	Transverse colon	G_Oscillibacter_RNT	2.22	2.44 × 10^–2^
CYP3A7	Transverse colon	G_Bacteroides_RNT	− 2.46	6.27 × 10^–6^
	Transverse colon	G_P_Proteobacteria_HB	2.28	7.96 × 10^–6^
	Transverse colon	G_Methanobrevibacter_HB	2.31	3.04 × 10^–5^
	Transverse colon	Div_Chao1_RNT	2.76	3.29 × 10^–5^
	Transverse colon	Div_NumberGenera_RNT	2.48	1.59 × 10^–4^
	Transverse colon	O_Burkholderiales_RNT	2.60	2.94 × 10^–4^
	Transverse colon	G_Victivallis_RNT	2.26	4.90 × 10^–4^
	Transverse colon	Div_Shannon_RNT	2.45	6.20 × 10^–4^
	Transverse colon	G_Gemmiger_HB	2.68	6.51 × 10^–4^
	Transverse colon	G_Methanobrevibacter_HB	2.19	9.41 × 10^–4^
WDR5B	Sigmoid colon	F_Veillonellaceae_HB	− 3.20	4.99 × 10^–4^
	Transverse colon	G_F_Porphyromonadaceae_RNT	3.31	2.00 × 10^–3^
	Sigmoid colon	G_F_Porphyromonadaceae_RNT	3.35	2.05 × 10^–3^
	Sigmoid colon	O_Rhodospirillales_HB	3.20	3.45 × 10^–3^
	Sigmoid colon	G_O_Clostridiales_RNT	2.37	3.57 × 10^–3^
	Sigmoid colon	C_Alphaproteobacteria_HB	3.37	3.91 × 10^–3^
	Sigmoid colon	G_P_Proteobacteria_RNT	− 2.51	4.66 × 10^–3^
	Transverse colon	F_Acidaminococcaceae_RNT	− 2.49	4.75 × 10^–3^
	Sigmoid colon	F_Rhodospirillaceae_HB	2.97	4.80 × 10^–3^
	Sigmoid colon	G_F_Rhodospirillaceae_HB	2.76	6.18 × 10^–3^
DFNB59	Sigmoid colon	G_F_Porphyromonadaceae_HB	− 3.67	7.08 × 10^–5^
	Sigmoid colon	G_Akkermansia_HB	− 2.64	6.56 × 10^–4^
	Sigmoid colon	F_Desulfovibrionaceae_RNT	− 3.30	8.33 × 10^–4^
	Sigmoid colon	G_Oscillibacter_RNT	− 3.87	8.78 × 10^–4^
	Sigmoid colon	G_Victivallis_HB	− 2.99	1.42 × 10^–3^
	Sigmoid colon	G_O_Clostridiales_RNT	− 3.13	1.97 × 10^–3^
	Sigmoid colon	G_F_Coriobacteriaceae_HB	− 2.99	2.54 × 10^–3^
	Sigmoid colon	G_Alistipes_RNT	− 2.85	2.91 × 10^–3^
	Sigmoid colon	G_Eisenbergiella_HB	− 2.12	3.39 × 10^–3^

### Fine mapping results

We performed fine mapping by FOCUS for 157 microbial traits with two reference panels, and finally found 11 genes included in 90%-credible sets, indicating the genes may causally associated with microbial traits (Table 3). Among them, 3 genes have been identified in TWAS analyses: HELLS for *Streptococcus* (RNT) (PIP = 0.685) in sigmoid colon, HELLS for *Streptococcaceae* (RNT) (PIP = 0.665) in sigmoid colon, ANO7 for *Erysipelotrichaceae* (RNT) (PIP = 0.449) in sigmoid colon, and STAG3L4 for *Lachnospiraceae* (RNT) (PIP = 0.171) in transverse colon.

**Table 3:** Potentially causal genes for microbial traits detected by FOCUS

Gene	Chrom	Microbial trait	Reference panel	PIP	Identified by TWAS
METTL15P1	3	G_Faecalitalea_RNT	Sigmoid colon	0.821	NO
COL5A1-AS1	9	G_Parasutterella_RNT	Sigmoid colon	0.801	NO
RP1-257A7.4	6	G_Veillonella_RNT	Sigmoid colon	0.714	NO
HELLS	10	G_Streptococcus_RNT	Sigmoid colon	0.685	YES
HELLS	10	F_Streptococcaceae_RNT	Sigmoid colon	0.665	YES
FBXO27	19	G_Aestuariispira_RNT	Sigmoid colon	0.555	NO
NIPSNAP1	22	G_Clostridium_sensu_RNT	Sigmoid colon	0.537	NO
ANO7	2	F_Erysipelotrichaceae_RNT	Sigmoid colon	0.449	YES
FRRS1L	9	G_P_Firmicutes_RNT	Transverse colon	0.826	NO
RP11-1277A3.2	5	G_Intestinibacter_RNT	Transverse colon	0.114	NO
STAG3L4	7	G_Lachnospiraceae_RNT	Transverse colon	0.171	YES
CPNE8	12	G_F_Porphyromonadaceae_RNT	Transverse colon	0.201	NO

The integral fine mapping results are shown in Additional file 5, 6: Table S5–S6

### Functional analyses results

The significant genes identified by TWAS for each microbial trait in the two tissues were subjected to functional analysis (Additional file 7: Table S7). Totally, we detected 94 GO terms in two phenotypes. For instance, GO_NUCLEOSIDE_DIPHOSPHATASE_ACTIVITY was significant for *Butyrivibrio* (RNT) (FDR *P* = 1.30 × 10^–4^), GO_CONDENSED_CHROMOSOME_CENTROMERIC_REGION was significantly associated with *Acidaminococcus* (HB) (FDR *P* = 1.17 × 10^–3^), GO_SPECTRIN_BINDING was detected to be correlated with *Burkholderiales* (RNT) (FDR *P* = 1.69 × 10^–3^), and GO_VACUOLE was associated with *Enterobacteriaceae* (RNT) (FDR *P* = 2.84 × 10^–3^).

FUMA also identified 11 pathways related to microbial traits, such as KEGG_RENIN_ANGIOTENSIN_SYSTEM for *Anaerostipes* (RNT) (FDR *P* = 3.16 × 10^–2^), KEGG_PURINE_METABOLISM for *Veillonellaceae* (HB) (FDR *P* = 7.35 × 10^–3^), KEGG_JAK_STAT_SIGNALING_PATHWAY for *Enterococcaceae* (RNT) (FDR *P* = 2.60 × 10^–2^). Table 4 shows the top 10 gene ontology terms and KEGG pathways of the significant genes.

**Table 4:** Top 10 significant GO and KEGG pathways for microbial traits

GeneSet	Microbial trait	FDR *P*
GO term		
GO_NUCLEOSIDE_DIPHOSPHATASE_ACTIVITY	G_Butyrivibrio_RNT	1.30 × 10^–4^
GO_CONDENSED_CHROMOSOME_CENTROMERIC_REGION	G_Acidaminococcus_HB	1.17 × 10^–3^
GO_KINETOCHORE	G_Acidaminococcus_HB	1.17 × 10^–3^
GO_CHROMOSOMAL_REGION	G_Acidaminococcus_HB	1.39 × 10^–3^
GO_SPECTRIN_BINDING	O_Burkholderiales_RNT	1.69 × 10^–3^
GO_CHROMOSOME_CENTROMERIC_REGION	G_Acidaminococcus_HB	2.55 × 10^–3^
GO_VACUOLE	F_Enterobacteriaceae_RNT	2.84 × 10^–3^
GO_OXIDOREDUCTASE_ACTIVITY_AC TING_ON_PAIRED_DONORS_WITH_INCORPORATION _OR_REDUCTION_OF_MOLECULAR_OXYGEN_REDUCED _FLAVIN_OR_FLAVOPROTEIN_AS_ONE_DONOR_AND _INCORPORATION_OF_ONE_ATOM_OF_OXYGEN	G_unclassified_F_Porphyromonadaceae_HB	3.62 × 10^–3^
GO_STEROID_HYDROXYLASE_ACTIVITY	G_unclassified_F_Porphyromonadaceae_HB	3.62 × 10^–3^
GO_CONDENSED_CHROMOSOME	G_unclassified_F_Porphyromonadaceae_HB	3.73 × 10^–3^
KEGG pathway		
KEGG_RETINOL_METABOLISM	G_unclassified_F_Porphyromonadaceae_HB	3.35 × 10^–3^
KEGG_METABOLISM_OF_XENOBIOTICS_BY_CYTOCHROME_P450	G_unclassified_F_Porphyromonadaceae_HB	3.35 × 10^–3^
KEGG_PURINE_METABOLISM	F_Veillonellaceae_HB	7.35 × 10^–3^
KEGG_METABOLISM_OF_XENOBIOTICS_BY_CYTOCHROME_P450	Div_Shannon_RNT	7.36 × 10^–3^
KEGG_FC_GAMMA_R_MEDIATED_PHAGOCYTOSIS	O_Selenomonadales_RNT	1.28 × 10^–2^
KEGG_NEUROACTIVE_LIGAND_RECEPTOR_INTERACTION	F_Desulfovibrionaceae_RNT	1.48 × 10^–2^
KEGG_PYRIMIDINE_METABOLISM	F_Veillonellaceae_HB	2.07 × 10^–2^
KEGG_JAK_STAT_SIGNALING_PATHWAY	F_Enterococcaceae_RNT	2.60 × 10^–2^
KEGG_LYSOSOME	F_Enterobacteriaceae_RNT	2.81 × 10^–2^
KEGG_RENIN_ANGIOTENSIN_SYSTEM	G_Anaerostipes_RNT	3.16 × 10^–2^

The integral functional analyses results are shown in Additional file 7: Table S7

### Association between candidate genes and diseases

The selected top genes in Tables 1 and 2 were searched on PubMed website to explore the possible relationship with diseases, and 12 genes were found to be associated with 12 diseases (Table 5). For instance, HELLS for *Streptococcus* in sigmoid colon was related to colorectal cancer [20], and SFTPD for an unclassified genus of *Proteobacteria* in transverse colon was detected to be related to atherosclerosis [21]. Specifically, although not included in the top genes, FUT2 for *Bifidobacterium* was suggested to be the causal gene for Crohn's disease (CD) in previous study [22].

**Table 5:** The list of candidate genes associated with diseases

Gene	Microbial trait	Reference panel	Gene-related disease	Reference
NDUFV3	Div_Chao1_RNT	Sigmoid colon	Down syndrome	PMID: 26848775
ARIH2	G_Coprococcus_HB	Transverse colon	Parkinson’s disease	PMID: 31284572
ZNF33A	G_P_Bacteroidetes_RNT	Transverse colon	Major depressive disorder	PMID: 32554045
ARHGAP1	C_Gammaproteobacteria_HB	Transverse colon	Ischemic heart disease	PMID: 31664016
CYP3A7	Div_Chao1_RNT	Transverse colon	Bilirubin metabolic disorder	PMID: 32499339
SFTPD	G_P_Proteobacteria_RNT	Transverse colon	Atherosclerosis	PMID: 26748346
LSG1	G_Streptococcus_RNT	Sigmoid colon	Attention-deficit hyperactivity disorder	PMID: 30738099
SH3PXD2B	F_Porphyromonadaceae_RNT	Sigmoid colon	Osteoporosis	PMID: 30962481
FUT2	G_Bifidobacterium_HB	Transverse colon	Crohn’s disease	PMID: 31260595
PKD1L2	G_Alloprevotella_RNT	Transverse colon	Colorectal cancer	PMID: 27605020
HELLS	G_Streptococcus_RNT	Sigmoid colon	Colorectal cancer	PMID: 32063710
ANO7	F_Erysipelotrichaceae_RNT	Sigmoid colon	Prostate cancer	PMID: 30157291

## Discussion

Host genes have been shown to be closely related to the ecosystem of the gut microbiota. Previous studies have detected multiple candidate genes associated with specific taxa [23–25]. Recent studies indicated that noncoding regulatory regions play an important role in influencing human complex traits. The gut microbiota was once suggested as a complex trait of the host affected by mbQTL [8], so we speculate that the host can influence the composition of the gut microbiota and the abundance of specific groups by regulating gene expression. In this study, TWAS was performed to prioritize candidate genes affecting gut microbiota at gene expression level by integrating GWAS summary data and specific pre-computed tissue expression profile. Finally, we identified numbers of genes and pathways related to microbial traits, and some of the genes have been reported to be associated with specific diseases by previous studies.

TWAS and fine mapping both prioritized several candidate genes for gut microbiota, such as HELLS for *Streptococcus* in sigmoid colon, ANO7 for *Erysipelotrichaceae* in sigmoid colon. We attempted to explore the relationship between gut microbiota candidate genes and diseases. HELLS encodes lymphoid specific, which participates in the establishment and maintenance of DNA methylation with chromatin remodeling through its ATPase activity [20]. HELLS expression was proved to be significantly associated with the colorectal cancer progression and a higher pathological grade [20]. Aberrant bands of the HELLS was observed in seven colorectal cancers by polymerase chain reaction-based single strand conformation polymorphism assay [26]. *Streptococcus* has been identified as colorectal cancer candidate pathogens in previous researches [27, 28]. ANO7 has been found to play a central role in prostate cancer progression, and its elevated expression correlates with disease severity and outcome [29]. Notably, the abundance of *Erysipelotrichaceae* was observed to be increased in prostate cancer patients [30]. In the treatment of prostate cancer by androgen axis targeted therapy, men receiving the treatment showed a significant decrease in the abundance of sequencing reads assigned to *Erysipelotrichaceae* [31]. In gut microbiota of mice, the abundance of *Erysipelotrichaceae* was also different between cancer bearing mice and healthy mice [32].

FUT2 was detected to be associated with *Bifidobacterium* in transverse colon in TWAS. FUT2 gene encodes α-1, 2-fucosyltransferase for the expression of ABH blood group antigens on mucosal surfaces, and determines the ability to secrete blood group antigens into gastrointestinal secretions. Individuals who have homozygous non-coding variants in FUT2 are nonsecretors, and ABH antigens are not expressed in mucosal secretions and surfaces, generally called as sese [33, 34]. Accordingly, secretory type was expressed as SeSe and Sese [34].

The alterations of FUT2 genotype resulted in a significant shift of microbial composition, that is, the gardening effect of FUT2 polymorphism on phylogenetic composition of the gut microbiota [34]. Present studies consistently show the genome-wide significant association between FUT2 non-secretor allele and CD in various races [22, 35]. It is suggested that FUT2 gene loss-of-function allele homozygotes change the gut microbiota of CD patients [36–39]. FUT2 polymorphism may also partly contribute to CD susceptibility by shaping community composition and structure of microbiota [36, 37]. Previous studies showed genus *Bifidobacterium* had higher diversity, richness and abundance in secretors compared with non-secretors [40, 41]. Moreover, increased genus *Bifidobacterium* is related to successful clinical outcome or remission of therapy in CD [42]. Further studies are warranted to identify the interactions between FUT2, *Bifidobacterium* and CD.

TWAS also identified SFTPD as a candidate gene for an unclassified genus of *Proteobacteria* in transverse colon. SFTPD encodes surfactant protein D, which is an important host defense lectin. It aggregates and enhances phagocytosis of microbes and dying host cells [43]. SFTPD is mainly expressed in lung, but also distributes in gallbladder and gut, and could shape intestinal microbial ecosystem [43]. Some potential evidence has carried out the link between SFTPD and phylum *Proteobacteria*. Nexoe et al., found a strong positive correlation between inflammatory activity and expression of SFTPD in the intestinal epithelium from Inflammatory Bowel Disease (IBD) patients [44], while the increase of *Proteobacteria* is one of the most consistent observations in IBD individuals [45].

SFTPD was reported exacerbating the development of atherosclerosis in previous literatures [21, 46–48]. In recent decades, bacterial infections and chronic inflammation have become possible causes of cardiovascular disease. Atherosclerosis is a chronic inflammatory process driven by lipids in the walls of the great arteries [49]. SFTPD has been proved to play a predominant role in pro-inflammatory [50, 51]. According to previous studies, the genus of *Proteobacteria* were involved in the formation of atherosclerosis. For instance, *Proteus vulgaris* was found to be present in the plaques and intestines of the same individual [52], *Proteus mirabilis* can interact with atherosclerosis plaques in human coronary arteries via specific molecular to exacerbate the progression of disease [53]. In addition, the abundance of *Proteus* in the blood of cardiovascular disease patients was observed to be increased compared with healthy individuals [52]. In mouse disease models, the reduction of phylum *Proteobacteria* abundance can exert a therapeutic effect on atherosclerosis [54]. Since the SFTPD is related to the abundance of bacteria from phylum Proteobacteria based on our findings, we hypothesized that the microbiota could affect susceptibility to atherosclerosis by genetic regulation.

KEGG_RENIN_ANGIOTENSIN_SYSTEM was detected to be associated with *Anaerostipes* in functional analysis. In a recent study, the fewer abundance of *Anaerostipes* was observed in primary aldosteronism patients than healthy individuals [55]. Bier et al. have confirmed that high salt diet could lead to decreased the abundance of taxa from the *Anaerostipes* genus [56]. Moreover, *Anaerostipes* was found to be correlated with higher estimated glomerular filtration rate in the overall population [57].

To the best of our knowledge, we conducted the first large-scale comprehensive sigmoid colon and transverse colon tissue-specific TWAS for gut microbiota, and performed fine mapping based on TWAS for further confirmation. The candidate genes for gut microbiota were further explored for the link between various taxa and diseases. Our study also has three potential limitations. First, only individuals of European ancestry from Germany and Belgium were included in the analysis, so the results cannot be generalized to other ethnic groups. Second, the information about diet and drug use of individuals is lack so that we can’t rule out the effects of diet and medication on the composition of gut microbiota. Third, it should be marked that the purpose of this study is to screen and prioritize candidate genes for gut microbiota, the results should be interpreted with caution. At present, research based on the interaction of genes and gut microbiota still needs more extensive exploration, further functional studies should be performed to confirm our findings and elucidate the mechanisms which genes act on gut microbiota.

## Conclusions

To be conclude, we performed TWAS analyses and identified multiple candidate genes and pathways of gut microbiota. We found that some candidate genes may also involve in the susceptibility of diseases, and attempted to provide clues for revealing the influence of genetic factors on gut microbiota for the occurrence and development of diseases. Our findings may provide new insight into the influence of genetic factors on the composition of gut microbiota, in addition to suggesting the potential role of gut microbiota in the mechanism of genetic factors contributing to disease susceptibility. Further studies are needed to demonstrate specific biological mechanisms in the future.

## Supplementary Information

**Additional file 1:** **Table S1.** The number of candidate genes for each microbial traits identified by TWAS.

**Additional file 2**: **Table S2.** TWAS results for gut microbiota in sigmoid colon.

**Additional file 3: Table S3. **TWAS results for gut microbiota in transverse colon.

**Additional file 4: Table S4.** Top 14 overlapped candidate gene for defferent microbial traits.

**Additional file 5: Table S5.** Fine mapping results for gut microbiota in sigmoid colon.

**Additional file 6: Table S6.** Fine mapping results for gut microbiota in transverse colon.

**Additional file 7: Table S7. **Functional analyses results for microbial traits.

## Data Availability

Raw 16S data used in the original GWAS study are available at the European Genome/ Phenome Archive under accession no. EGAS00001004420. The microbiome GWAS summary data support the findings of this study are available online at the University of Bristol data repository with the identifier https://doi.org/10.5523/bris.22bqn399f9i432q56gt3wfhzlc. The precomputed expression weights of tissues derived from the genotype-Tissue expression (GTEx) project were downloaded from FUSION websites http://gusevlab.org/projects/fusion/. The TWAS results of this study have been deposited in the Figshare repository at the following http://dx.doi.org/10.6084/m9.figshare.14387162.
